# Soft, Biological and Composite Nanomaterials

**DOI:** 10.3390/nano10081488

**Published:** 2020-07-29

**Authors:** Arvind Gupta, Beom Soo Kim

**Affiliations:** Department of Chemical Engineering, Chungbuk National University, Cheongju, Chungbuk 28644, Korea; arvind@chungbuk.ac.kr

The progress in the area of nanotechnology has opened the door for the fabrication of soft, biological and composite nanomaterials for targeted applications. Nanomaterials are known to enhance the properties and functionality of composite materials severalfold. The properties for the desired applications can often be achieved by the addition of small amounts of nanomaterials into soft materials such as polymers, gels, and biomaterials. Various techniques such as the functionalization of nanomaterials and the fabrication of composites in situ are ground-breaking methods that may lead to a significant improvement in the properties of these materials. Furthermore, there is a need for a focused characterization of the developed materials in order to use them for targeted applications, which will ultimately contribute to the future development of nanomaterials and their composites for various applications. Nanomaterials, such as nanoparticles and graphene, are also found to have a tremendous potential for a wide variety of biomedical applications such as antimicrobial and antitumor agents, drug delivery, tissue engineering, biosensors, bioimaging, and enzyme mimics. Therefore, there is a growing need to develop environmentally friendly processes for nanomaterial synthesis, such as biological methods using microorganisms, enzymes, and plants/plant extracts.

The current special issue of nanomaterials features an overview of several articles (eight research articles and one review article), wherein the use of various nanomaterials such as nanodiamonds, gold nanoparticles, nanochitosan, graphene oxide nanoparticles, and titanium nanofibers have been shown.

The use of nanodiamonds (NDs) for the fabrication of scaffolds may be effective in tissue regeneration. The inherent properties of NDs, such as a relatively low cytotoxicity, fluorescence, large specific surface area, and hardness, render them as potential nanomaterials for composite tissue engineering scaffolds. The incorporation of NDs (45 nm) as fillers in a well-known biocompatible polymer, i.e., poly(ε-caprolactone) (PCL) was investigated by Fox et al. [1] who observed that the composites possess biocompatibility and better degradation properties along with processability. The presence of NDs imparts roughness to the surface of PCL, thereby improving its hydrophilicity and promoting the degradation rate with a compromise on its tensile strength. An enhanced adhesion of osteoblast cells to the composite in comparison to the virgin PCL paves the way for the development of advanced tissue engineering scaffolds. Further, Nirwan et al. [2] fabricated a material using a neutralized chitosan/polyethylene oxide-based nanofiber decorated with gold nanoparticles for the development of tissue engineering scaffolds via an electrospinning technique. They proposed a simple neutralization process in order to conserve the structural integrity of the fabricated nanofibers over a period of six months. The neutralization of the reactive NH_3_^+^ group using potassium carbonate and transforming it into NH_2_ makes this material insoluble in the biological system. This enhances the mechanical integrity of the product over six months in the biological solution and renders it suitable for tissue engineering applications.

Nanoceria as a nanomaterial can be used for the detection of galactose, which is an important marker for the diagnosis of galactosemia. Nguyen et al. [3] developed a composite of ceria oxide nanoparticles and galactose oxidase entrapped in an agarose gel, and this was found to be capable of detecting galactose. The enzymatic catalysis of galactose generates hydrogen peroxide (H_2_O_2_) which induces a yellow color in the system without any chromogenic substrate. The calibration of the generated color using an electronic system can detect galactose with a sensitivity as low as 0.05 mM.

Further, graphene oxide (GO) which is known for its unique mechanical, thermal, optical, and electrical characteristics, is found to have a potential use in biomedical applications, apart from its widespread use in electronics and chemical applications. The different sizes of GO nanoparticles may have different effects on cell proliferation. Gurunathan et al. [4] conducted a study to understand the cyto- and geno-toxic effect of GO nanoparticles on germ (TM3 and TM4) cells. They concluded that both 20 nm and 100 nm sized GO nanoparticles exert a potent cytotoxic effect by reducing cell viability and proliferation. They found that the smaller sized (20 nm) GO has a more negative effect than 100 nm GO. Through this study, they revealed that GO is not 100% safe for biomedical applications and a thorough investigation is required, even though GO has the potential to be used for biomedical applications.

For other areas of biomedical applications where the shape memory of a polymer is predominantly required, Gupta et al. [5] developed polyurethane biocomposites. They found a way to utilize chitosan for the fabrication of biocomposites. Usually, chitosan is not compatible with hydrophobic polymers such as PCL. They transformed chitosan flakes to nanochitosan using a chemical treatment and incorporated them into the polyurethane matrix in situ in such a way that they can form a chemical linkage. The presence of nanochitosan in the polyurethane matrix acted as a chemical crosslinked node that governed the crystallinity of the biocomposite, thereby stimulating the shape memory with enhanced mechanical properties.

Another area of research in biomedical applications is drug delivery systems. The use of micro and nanostructures can be exploited for diagnostic and therapeutic applications. Nanobubbles, which can consist of a shell made of phospholipids, polymers, or proteins surrounding the core of a less soluble gas, are utilized for gas delivery applications. Khan et al. [6] aimed at identifying the role of the composition and use of polyethylene glycol (PEG) as a surfactant in the development of oxygen nanobubbles. They found that an increase in the content of PEG leads to a reduction in the nanobubble size and its distribution. The use of the generated oxygen nanobubbles was found to be non-toxic and did not cause hemolysis in sheep blood. Oxygen nanobubbles were also employed in an ultrasound imaging technique and were found to be traceable. Therefore, oxygen nanobubbles can be used for gas delivery applications and ultrasound imaging as well.

The controlled mechanism of the drug release rate is an important parameter in drug release or delivery systems. The use of magnetic nanoparticles can be effective in this application because it allows external stimulation. Mierzwa et al. [7] developed a biocompatible hybrid cubical phase nanomaterial called magnetocubosomes, containing hydrophobic magnetic nanoparticles which allow for the control of the release of the drug methotrexate at appropriate sites. It can be easily separated or relocated under the influence of magnetic fields.

Nanomaterials have become extensively important in photovoltaic applications as well. Titanium oxides (TiO_2_) nanoparticles can be used in dye-sensitized solar cells (DSSCs) as a transparent conductive layer. Jo et al. [8] used an electrospinning technique to fabricate porous and dense TiO_2_ nanofibers with 200 nm diameters as an additive in TiO_2_ nanoparticles for DSSCs. They found that the incorporation of nanofibers enhances the performance of DSSCs by improving the charge transport and accessibility to electrolyte ions. It also enhanced the absorption of visible light in comparison to TiO_2_ nanoparticles.

Furthermore, this special issue contains a review article by Perepelkin et al. [9] which focuses on different depth-sensing indentation (DSI) approaches and factors to characterize the mechanical properties of soft, biological, and biomimetic materials at the micro- and nanoscale.

Overall, this special issue highlights investigations in highly diverse research fields to encourage multifaceted research in the scientific community.

## References

[1] Fox K., Ratwatte R., Booth M.A., Tran H.M., Tran P.A. (2020). High Nanodiamond Content-PCL Composite for Tissue Engineering Scaffolds. Nanomaterials.

[2] Nirwan V.P., Al-Kattan A., Fahmi A., Kabashin A.V. (2019). Fabrication of Stable Nanofiber Matrices for Tissue Engineering via Electrospinning of Bare Laser-Synthesized Au Nanoparticles in Solutions of High Molecular Weight Chitosan. Nanomaterials.

[3] Nguyen P.T., Ahn H.T., Kim M.I. (2020). Reagent-Free Colorimetric Assay for Galactose Using Agarose Gel Entrapping Nanoceria and Galactose Oxidase. Nanomaterials.

[4] Gurunathan S., Kang M.H., Jeyaraj M., Kim J.H. (2019). Differential Cytotoxicity of Different Sizes of Graphene Oxide Nanoparticles in Leydig (TM3) and Sertoli (TM4) Cells. Nanomaterials.

[5] Gupta A., Kim B.S. (2019). Shape Memory Polyurethane Biocomposites Based on Toughened Polycaprolactone Promoted by Nano-Chitosan. Nanomaterials.

[6] Khan M.S., Hwang J., Lee K., Choi Y., Jang J., Kwon Y., Hong J.W., Choi J. (2019). Surface Composition and Preparation Method for Oxygen Nanobubbles for Drug Delivery and Ultrasound Imaging Applications. Nanomaterials.

[7] Mierzwa M., Cytryniak A., Krysiński P., Bilewicz R. (2019). Lipidic Liquid Crystalline Cubic Phases and Magnetocubosomes as Methotrexate Carriers. Nanomaterials.

[8] Jo M.S., Cho J.S., Wang X.L., Jin E.M., Jeong S.M., Kang D.W. (2019). Improving of the Photovoltaic Characteristics of Dye-Sensitized Solar Cells Using a Photoelectrode with Electrospun Porous TiO_2_ Nanofibers. Nanomaterials.

[9] Perepelkin N.V., Borodich F.M., Kovalev A.E., Gorb S.N. (2020). Depth-Sensing Indentation as a Micro- and Nanomechanical Approach to Characterisation of Mechanical Properties of Soft, Biological, and Biomimetic Materials. Nanomaterials.

